# Emergence of microbial resistance against nanoparticles: Mechanisms and strategies

**DOI:** 10.3389/fmicb.2023.1102615

**Published:** 2023-01-26

**Authors:** Siya Kamat, Madhuree Kumari

**Affiliations:** Department of Biochemistry, Indian Institute of Science, Bangalore, India

**Keywords:** nanoparticles, resistance, microbes, flagellin, selection pressure

## Abstract

Antimicrobial nanoparticles have gained the status of a new generation of drugs that can kill bacterial pathogens by multiple means; however, nanoparticle resistance acquired by some bacterial pathogens has evoked a cause of concern. Several reports suggested that bacteria can develop nanoparticles, specifically metal nanoparticle resistance, by mechanisms: nanoparticle transformation-induced oxidative stress, membrane alterations, reversible adaptive resistance, irreversible modifications to cell division, and a change in bacterial motility and resistance. Surface properties, concentration and aggregation of nanoparticles, biofilm forming and metal exclusion capacity, and R plasmid and flagellin synthesis by bacteria are crucial factors in the development of nanoparticle resistance in bacteria. Studies reported the resistance reversal by modifying the surface corona of nanoparticles or inhibiting flagellin production by bacterial pathogens. Furthermore, strict regulation regarding the use and disposal of nano-waste across the globe, the firm knowledge of microbe–nanoparticle interaction, and the regulated disposal of nanoparticles in soil and water is required to prevent microbes from developing nanoparticle resistance.

## Introduction

One of the biggest public health concerns of the 21^st^ century is growing antimicrobial resistance (Murray et al., 2022). The “at-risk” group includes patients with underlying health problems or compromised immune systems such as HIV, cancer, diabetes, respiratory disorders, autoimmune diseases, or simply old age. The World Health Organization (WHO)^1^ has recognized this threat and started a worldwide initiative of creating awareness and understanding of antimicrobial resistance, surveillance, and research to strengthen knowledge, reduce the incidence of microbial infection, optimized the use of antimicrobial drugs, and development of the economic case of sustainable investment in tackling the issue. Early reports focused exclusively on resistant bacterial pathogens. The most prevalent examples include methicillin-resistant *Staphylococcus aureus*, chloroquine-resistant *Plasmodium falciparum*, and fluconazole-resistant *Candida albicans*.

Penicillin, the first antibiotic, was discovered from *Penicillium notatum* in 1928. Following this revolutionary discovery, several other natural sources, such as plants (Vaou et al., 2021), endophytic fungi (Kamat et al., 2022b), marine organisms (Kamat et al., 2020a; Sajna et al., 2020), and lichens (Kumari et al., 2022) have been explored for natural products with therapeutic properties (Kamat et al., 2020b). Griseofulvin derivatives are examples of naturally occurring antibiotics commonly produced by *P. griseofulvum* (Zhang et al., 2017). Recent discoveries in antimicrobial therapeutics have focused on the potential of antimicrobial peptides such as defensins, tachyplesin, histatin, and magainin (Magana et al., 2020). Unfortunately, the discovery or development of new antimicrobial drugs is unable to catch up with the emerging antimicrobial resistance (Huh and Kwon, 2011). Nanomaterials have received considerable attention for being effective antimicrobials. They include nanosystems such as lipid-based nanoparticles (liposomes, niosomes, and solid/nanolipid carries), polymer-based nanoparticles (dendrimers, nanocapsules, nanofibers, and nanospheres), metallic or inorganic nanoparticles (gold/silver nanoparticles), carbon-based (graphene, fullerenes, carbon dots, carbon rings, etc.), and nanozymes (Raza et al., 2021; Vanić et al., 2021). To design new therapeutic interventions against multidrug-resistant *Mycobacterium tuberculosis*, Sheikhpour et al. (2022) synthesized fluoxetine and an isoniazid-conjugated multi-walled carbon nanotube nanofluid. The new nanosystem was effective at low concentrations in treating clinical strains of *M. tuberculosis*. A novel photodynamic nanoformulation composed of chlorin e6 (Ce6)-loaded water-soluble chitosan-coated iron oxide nanoparticles demonstrated bactericidal activity and biofilm penetration ability against methicillin-resistant *Staphylococcus aureus* (Jin et al., 2022). Conjugation of nanoparticles with potent antimicrobial small molecules such as peptides and antibiotics has also been explored to further the versatility of nanomaterials and mitigate emerging antimicrobial resistance to antibiotics (Kollef et al., 2011). The nanoparticle itself serves as an antimicrobial partner or a targeting agent in the system. Jelinkova et al. (2019) have summarized the different combinations of nanoparticles and antibacterial agents and strategies to synthesize them. Silver, gold, zinc oxide, copper, and nanoparticles that have been conjugated with fusidic acid, gentamycin, ciprofloxacin, and penicillin are a few of these. Single-walled or multi-walled carbon nanotubes functionalized with nisin antimicrobial peptide, porphyrin, have been synthesized. Fullerenes, functionalized with isoniazid, quinazolin, vancomycin, or other antimicrobial peptides have been investigated against *Mycobacterium tuberculosis, Salmonella typhimurium*, and *Staphylococcus aureus*. Graphitic carbon dots, graphene oxide-based, penicillium-based, and citric acid-based carbon dots were conjugated with penicillin, ciprofloxacin, and vancomycin and tested against several multidrug-resistant bacterial pathogens. Conjugated nanoparticles have demonstrated higher efficiency in their bactericidal activity through mechanisms such as DNA and RNA damage, disruption in membrane architecture and integrity, oxidative stress, disruption of efflux pumps, and respiratory complexes. The nano part of this system ensures better penetration of the antimicrobial peptide or small molecule due to its charges, chemistry, and electron density. The metal-conjugated systems such as silver, gold, and nanoparticles also provide a stable coordination link and an enhanced antibacterial activity due to synergistic effect. Although promising, the exact mechanism of these nano-conjugated systems needs to be traced (Jelinkova et al., 2019; Gatadi et al., 2021).

Clearly, nanotechnology has immense potential and ubiquity in fighting microbial pathogens and several reviews have highlighted its value (Zhu et al., 2014; Raza et al., 2019; Eleraky et al., 2020; Fan et al., 2021; Rubey and Brenner, 2021; Vanić et al., 2021). The antimicrobial property of silver nanoparticles has galvanized the healthcare sector wherein nanoparticles form the bactericidal coatings in medical devices. They are also found in cosmetics, textiles, and packaging materials (Khan et al., 2019). Resistance to silver ions has already been reported in several pathogenic bacteria. Unfortunately, recent studies revealed that the chemical versatility of nanoparticles has also contributed to the emergence of microbial resistance (McNeilly et al., 2021; Yonathan et al., 2022).

In this review, we intend to shed light on the developing nanoparticle-resistant microbial pathogens that can become a major threat to human health. We propose the necessity of synergistic approaches in developing nanomaterial-based antimicrobials with long-term benefits.

## Microbial resistance against nanoparticles

Metallic or inorganic nanoparticles and nanocomposites have demonstrated great promise in medical and biotechnological applications due to their intrinsic antimicrobial activity. Silver, copper, gold, silica, iron oxide, titanium dioxide, and nanoparticles are commonly found as antimicrobials in surface coating, textile industry, wound dressings, food preservation, water treatment, and cosmetics and have been anticipated in drug delivery applications (Khan, 2020). There have been attempts at several approaches to predict the impact and behavior of a microbial population exposed to nanoparticles. An important consideration in these experiments is the environmental pH, salts, shape, size and chemistry of nanoparticles, and external medium. Even a small change in these conditions or parameters can impact the microbe–nanoparticle interactions (Joshi et al., 2012; Ewunkem et al., 2021). Through experimental evolution studies, it is now known that bacterial pathogens can counter antimicrobial nanoparticles. Table 1 summarizes some of the examples of nanoparticle resistance developed in microbes.

**Table 1:** Examples of microbial resistance to antimicrobial nanoparticles.

**Type of nanoparticle**	**Resistant organism**	**Resistance developed after how many generations or days of evolution**	**Genetic/cellular/phenotypic changes observed**	**Reference**
Citrate coated Ag nanoparticles	*E. coli* K-12 MG1655	225 generations	Mutation in *cusS, purl, rpoB, ompR*	Graves et al., 2015
Ag nanoparticles	*E. coli*/BW25113/*ΔyhaK*	-	Overproduction of exopolysaccharides	Joshi et al., 2012
Ag nanoparticles	*E.coli* 013, *Pseudomonas aeruginosa* CCM 3955 and *E. coli* CCM 3954	-	Production of adhesive flagellum protein flagellin triggering nanoparticle aggregation	Panáček et al., 2018
Ag_2_S coated Ag nanoparticles	*E. coli*	>200 days	Upregulation of MDR genes, Cu efflux transporter genes	Li et al., 2019
Magnetite nanoparticles	*E. coli*	25 days of selection	Increased cell length, selective sweeps in *rpoA* and *rpoC*	Ewunkem et al., 2021
ZnO nanoparticles	*E. coli*	25 generations	Changes in cell shape-rod to oval probably due to low expression of membrane protein RodZ, porins	Zhang et al., 2018
Ag nanoparticles	Model microbiota of environmental or clinical setting: *E. coli* and *Bacillus* sp.	-	Modulation of Z-ring division septum, upregulation of cytoprotective genes-permease components, efflux proteins	Gunawan et al., 2013
OH- functionalized single walled carbon nanotubes	*E. coli*	90 min	Elevated expression of *pspA* and *pspC* when exposed to high levels of carbon nanotubes	Anh Le et al., 2019
PbS nanoparticles	*Saccharomyces cerevisiae*	-	Increased chitin deposition in the cell wall and activated cell wall integrity pathway genes (*FKS2* and *PRM5*)	Sun et al., 2014
Nanoscale zerovalent iron (nZVI)	*Pseudomonas putida F1*	-	Rigid cell membrane due to *cis* to *trans* conversion of unsaturated fatty acids	Kotchaplai et al., 2017

## Mechanisms of nanoparticle resistance

**Figure 1:**
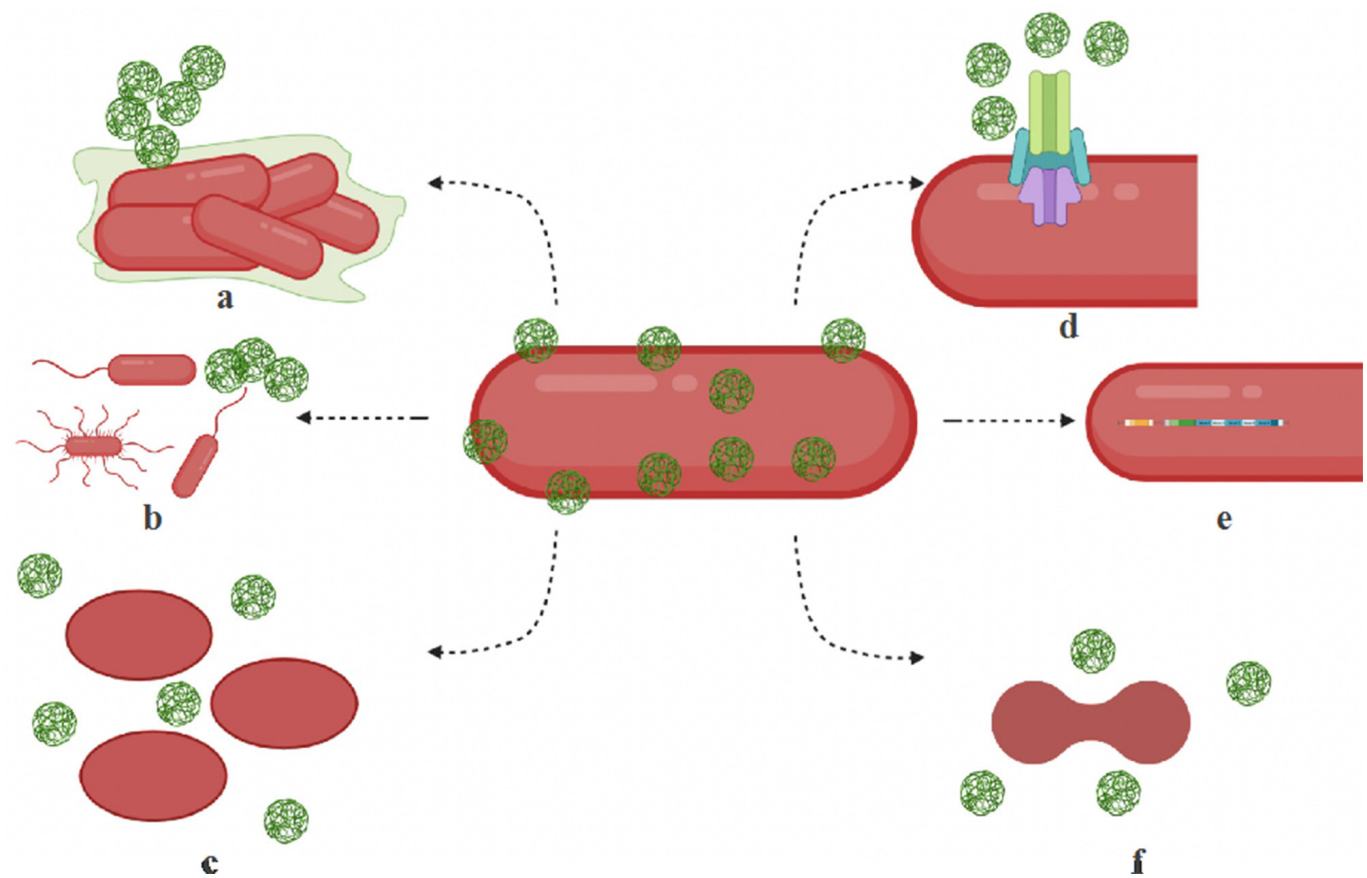
Mechanisms of microbial resistance to nanoparticles. **(a)** Biofilm — bacteria produce exopolysaccharides, biofilm to protect themselves or to aggregate nanoparticles. **(b)** Motility structures — hypermotile bacteria can evade nanoparticles and increase nutrient availability. **(c)** Shape change — bacteria switch shapes (rods to oval) by the isomerization of fatty acids, membrane lipids, and proteins and filter out nanoparticles. **(d)** Efflux systems — resistant microbes are known to overproduce efflux complex systems. **(e)** Operons — activation specialized operons and genes make activate cytoprotective mechanisms in bacteria. **(f)** Cell division disruption — nanoparticle stress can disrupt cell cycle regulation contribute to its resistance.

Microbial adaptation to nanoparticles has been observed through efflux pumps, electrostatic repulsion, biofilms and other extracellular polymeric substances, enzyme detoxification, volatilization, and genetic changes (Salas Orozco et al., 2019; Raza et al., 2021). Nanoparticles also undergo transformation in natural environments (Li et al., 2019). Figure 1 illustrates some of the common mechanisms of nanoparticle resistance developed in microbes. The key molecular players that drive the resistance mechanisms are summarized in Table 2. In this section, we will summarize the mechanisms of nanoparticle resistance adopted by microbes.

**Table 2:** Nanoparticle-resistance mechanisms adapted by bacterial pathogens.

**Resistance mechanism**	**Key cellular and molecular players**	**Reference**
Nanoparticle transformation-induced oxidative stress	Upregulation of efflux genes: *cusFCBA, marA, acrAB-tolC*	Li et al., 2019
Membrane alterations	Membrane vesicles	Pagnout et al., 2021
Reversible adaptive resistance	Change in cell shape by dysregulation of membrane porin proteins (OmpC, OmpF), RodZ, TolC, SoxS	Zhang et al., 2018; Pagnout et al., 2021
Irreversible modifications to cell division	Oxidative stress induced SOS response upregulates cell protective proteins YpsA, UgtP. This results in impaired FtsZ assembly causing inhibition of cell division.	Gunawan et al., 2013
Motility	Upregulation of *sil* operon that encodes efflux pumps. Non-genetic change: flagellin and biofilm.	Silver, 2003; Stabryla et al., 2021
Heritable resistance	Single nucleotide variation in *purR* gene resulting in overproduction of purines. Single nucleotide deletion in the *tcyA* gene reducing cysteine-mediated redox imbalance.	Even et al., 2006; Valentin et al., 2020

### Nanoparticle transformation-induced oxidative stress

Silver nanoparticles are the most widely used in commercial applications such as personal care products. They are highly stable and can persist in the natural environment resulting in chronic exposure threats. The common Ag nanoparticles' physicochemical transformations include aggregation, dissolution, oxidation, sulfidation, and organic coating; sulfidation being the key mechanism of transformation (Levard et al., 2012). *E. coli* chronically exposed to silver sulfide nanoparticles accumulated phenotypic and genotypic changes that contributed to its tolerance and resistance to various antibiotics. Chronic exposure to silver sulfide nanoparticles causes an upregulation of stress-related genes belonging to electron transport, cellular respiration, indicated respiration pathway disturbances, and oxidative stress. Upregulation of the multicomponent copper transport system (*cusFCBA*) which belongs to the P-type ATPases correlated well with the increase in energy requirement. The authors reported that this nanoparticle-induced intracellular stress also caused an upregulation of the multidrug-resistant genes. These changes triggered decreased sensitivity to antibiotics such as penicillin, kanamycin, ciprofloxacin, and gentamycin. Thus, oxidative stress started a domino effect affecting the overexpression of membrane porin genes and MDR efflux genes such as *marA* and *acrAB-tolC*, thus promoting adaptive pathogenic evolution (Li et al., 2019). This study highlighted the necessity to evaluate the sulfidation of nanoparticles or any modifications to the surface chemistry of nanoparticles for improved bactericidal activity, as it can backfire.

### Membrane alterations

Nanoparticles first encounter the microbial cell membrane. Therefore, the nanoparticle-induced biophysical and biochemical changes need to be understood to determine toxicity or tolerance. The lipids and proteins in the cell membrane are highly dynamic and are altered in response to any environmental stress. These changes can serve as a fingerprint to understand the biophysical mechanism of nanoparticles (Kamat et al., 2022a). The role of bacterial cell membrane composition and the toxicity mechanisms of metal oxide nanoparticles are currently debated. Recent studies attempted to decipher this relation using transcriptomics, nanomechanics, electrodynamics, and fluorescence assays (Pagnout et al., 2021). The effect of commercial nanoscale zerovalent iron on nanoparticle–membrane interaction was evaluated in *P. putida* F1. Beyond a certain concentration, the tolerance to nanoparticles increased substantially. The nanoparticles accumulated onto and penetrated the bacterial cell membrane, making it rigid due to cis/trans isomerization of fatty acids. The tolerant bacterial cells have a higher charge density and lower electrophoretic softness possibly due to modification in outer membrane components (Kotchaplai et al., 2017). Since metal oxide nanoparticles have effective antibacterial properties, their toxicity mechanisms are studied in detail. For a long time, the antibacterial activity of TiO_2_ nanoparticles was associated with its photocatalytic property that could induce ROS, membrane alteration, and lipid peroxidation. However, the same observations were also recorded even in the absence of light for TiO_2_ nanoparticles and other nanomaterials such as ZnO, MgO, CeO_2_, and fullerene nanoparticles. The toxicity mechanisms in dark were associated with osmotic stress and cell membrane alterations as a result of electrostatic attachment of metal nanoparticles as well as mechanical membrane disruption (Pagnout et al., 2012; Leung et al., 2016). Such studies highlighted the necessity to unravel the interaction of nanoparticles and membrane proteins, lipopolysaccharides, and membrane composition that could govern the possible entry of metallic nanoparticles into the cell. The differential sensitivity of bacteria to TiO_2_ nanoparticles was studied by Pagnout et al. (2021), wherein they specifically focused on the much-neglected membrane vesicles. Since the production of membrane vesicles is utilized as a defense in mitigating oxidative or osmotic stress, they could have an implication in nanoparticle resistance. The authors exposed *E. coli* K-12 mutant strains with truncated lipopolysaccharide (LPS) and *rfaC* gene mutation to TiO_2_ nanoparticles and recorded the resistance or sensitivity. These bacteria with altered membranes could efficiently resist the toxicity of TiO_2_ nanoparticles its hypervesiculation capacity and regulation of osmotic stress. With an increasing concentration of TiO_2_ nanoparticles, the membrane integrity is compromised coupled with oxidative stress and lipid peroxidation. There is also an increase in the secreted membrane vesicles to cope with the toxicity. They could be mediating membrane stress relief through the shuttling of proteins, LPS, and other harmful peroxidation products that accumulate in the periplasmic space or cause modifications on the outer membrane (Pagnout et al., 2021). This study warrants the consideration of membrane biophysics and composition to evaluate the sensitivity of nanoparticles.

### Reversible adaptive resistance

Zhang et al. (2018) evaluated the effect of ZnO nanoparticles in *E. coli*. The resistant bacteria could grow at high concentrations of ZnO (1,000 μg/mL). When the nanoparticles were removed from the growth medium for several days, the antibacterial sensitivity was restored. The development of this transient resistance is adaptive and not due to genetic changes but basically due to changes in the shape of bacteria. The resistant bacteria were oval shaped and switched to the typical rod-like shape when turned sensitive. The authors suggested this change in shape could be due to the lack of protein RodZ or envZ that provides binding sites on the cell membrane for cytoskeletal protein MreB. It is anticipated that the bacteria could have adapted to the low expression of RodZ and the high concentration of ZnO led to the oval phenotype. Another possible mechanism could be related to the porin proteins OmpC and OmpF. While OmpC is more restrictive than OmpF, it is the ratio of these porins that is influenced by the nanoparticles. ZnO nanoparticles could be binding to cAMP, thereby blocking the formation of the cAMP–CRP complex that would otherwise bind to the DNA for the production of OmpR. The resulting low expression of OmpR results in limited phosphorylation of OmpR which, in turn, will increase the expression of OmpF. This results in a more permeable outer membrane as OmpF is an open porin complex (Forst et al., 1988). Because the number of porins per cell volume increases when the cell shape changes to oval, it provides an additional route for nano resistance by mere change in the expression of OmpF when stressed by antibacterial nanoparticles. The authors conjecture that the oval shape during prolonged exposure to nanoparticles reduces the possibility of the nanoparticles entering the bacteria and therefore contributes to unstable nanoparticle resistance (Zhang et al., 2018). We infer that it is the change in membrane proteins such as the porins and RodZ that renders the bacteria momentarily resistant to ZnO nanoparticles. This is because, the porins, especially the OmpF allow the passive transport of small solutes. The nanoparticles could be crossing the bacterial membrane but a different mechanism allows for survival. Pagnout et al. (2021) observed non-monotonous OmpF levels with varying concentrations of TiO_2_ nanoparticles. The increased membrane permeability compensates for the Turgor stress and allows the survival of bacteria. In a recent study, the biocidal effect of nanostructured anatase rutile and carbon coating was observed to induce adaptive resistance in *E. coli* BW25113 (Wasa et al., 2022). The bacteria were observed to utilize their innate response to toxic environments that included changes in efflux proteins and membrane permeability. An increase in the levels of outer membrane protein TolC and superoxide stress protein SoxS was recorded; however, a whole transcriptome analysis is necessary to trace a detailed map of the mechanism.

### Irreversible modifications to cell division

Bacteria can adapt to environmental conditions by regulating cell division and external conditions too can modulate cell division. This is a well-orchestrated event and involves the transduction of environmental signals to control cell division proteins. In most bacteria, FtsZ is the central tubulin-like protein that marks the site of cytokinesis. Nanoparticle-induced oxidative stress initiates an SOS response and an upregulation of protective proteins such as YpsA and UgtP. Overproduction of such proteins leads to impaired FtsZ ring assembly and ultimately cell division inhibition (Brzozowski et al., 2019). Prolonged exposure to nanoparticle stress could induce an irreversible change in the tightly regulated cell division cycle “Z-ring component assembly” (Gunawan et al., 2013). In bacteria, several regulatory proteins render the Z-ring assembly/disassembly dynamic. During an SOS response, a regulatory protein *ugtP* delays cell division by destabilizing the Z-ring (Adams and Errington, 2009). This halts the cytokinesis and therefore the cell division cycle stops. This mechanism could be an indirect protective mechanism developed by bacteria against nanoparticles and needs to be further investigated.

### Motility and resistance

Stabryla et al. (2021) evaluated whether Ag nanoparticles or the released Ag(I) ions or their combination drives the resistance in *E. coli*. Bacterial resistance to Ag(I) ions through efflux pumps or reduction to its lesser toxic oxidation state Ag(0) is well established. The resistance is also attributed to *E. coli* deficiency in outer membrane proteins, resulting in the limited uptake of Ag. The *sil* operon in gram-negative bacteria is also well studied and encodes for efflux pumps: *SilP* (P-type ATPase) and *SilCBA* (antiporter) along with SilE and SilF (two periplasmic Ag binding chaperones. These components function synergistically to drive out Ag(I) of the gram-negative bacteria (Silver, 2003). A hypermotile *E. coli* strain demonstrated specific resistance to Ag nanoparticles as compared to the non-motile phenotypic strain. Both strains demonstrated the same degree of particle aggregation, indicating that aggregation alone could not be responsible for the differential resistance. However, an alternative mechanism that could be responsible is that the motile bacteria relocate away from the aggregated nanoparticles, thus reducing exposure to nanoparticles and increasing nutrient availability (Stabryla et al., 2021). Flagellin also triggers the aggregation of nanoparticles and contributes to resistance in *P. aeruginosa* CCM 3955 and *E. coli* CCM 3954. The evolution of this non-genetic change reduces the colloidal stability of nanoparticles and their antibacterial property. Flagellin forms the extracellular flagellar filaments necessary for bacterial motility. It also has adhesive properties and together with flagellum is critical for biofilm formation. However, the addition of pomegranate rind extract (which inhibits flagellin production) eliminated nanoparticle aggregation and bacterial resistance to Ag nanoparticles (Panáček et al., 2018).

### Heritable resistance

Valentin et al. (2020) reported the development of heritable adaptation to nanosilver (NAg) in *S. aureus* in an extensive study. The bacterium was serially cultured in the presence or absence of nanosilver for 50 days as performed for antibiotic resistance development (Ling et al., 2015). Whole-genome sequencing of nanoparticle-adapted *S. aureus*, the unevolved wild-type *S. aureus* and the wild-type *S. aureus* revealed two critical clues that explained the heritability of the evolved resistance adaptations. The first difference observed in Nag-resistant *S. aureus* was a single-nucleotide variation (TCT to TAT mutation) in the *purR* gene that encodes PurR, a putative purine operon repressor. The mutation resulted in serine to tyrosine at position 169 in the binding motif of PurR. PurR binding to the operon generally downregulates the enzymes responsible for the synthesis of inosine monophosphate (a precursor of the adenosine and guanosine monophosphates) (Cui et al., 2013). The evolved mutation reduces the binding affinity of the repressor resulting in elevated purine biosynthesis. Since NAg induces bacterial toxicity by DNA and protein damage, the evolved resistance mechanism compensates for survival by the overproduction of purines necessary for DNA replication, repair, and amino acid synthesis. The second difference observed was a single-nucleotide deletion in the *tcyA* gene of the Nag-resistant *S. aureus*. The gene encodes TcyA, a putative L-cystine binding protein that imports cystine from the extracellular environment into the cytoplasm for reduction to cysteine. In the bacterium, cysteine contributes to the iron–sulfur active site of thioredoxin and many enzymes of the tricarboxylic acid cycle. The oxidative stress induced by NAg exposure is mitigated by the antioxidant action of thioredoxin. The ATC to -TC mutation in the *tcyA* translates into a truncated protein. This results in a reduced import of cystine and low cellular cysteine (Even et al., 2006). This could reduce the cysteine-mediated redox imbalance linked to NAg toxicity in bacterial cells. These mutations were continually detected in daughter cells even upon removal of NAg exposure (Even et al., 2006; Valentin et al., 2020).

## Factors affecting the nanoparticle resistance in microbes

Multiple features of microbes and nanoparticles can impact the resistance development in the microbes which are discussed later:

### Type, size, geometry, and surface properties of nanomaterials

The most important parameters which affect the antimicrobial properties of nanoparticles are their type, shape, size, and the type of surface corona (Kumari et al., 2016, 2017). The maximum number of cases of microbes developing nanoparticle resistance is against metal nanoparticles, specifically silver and copper nanoparticles (Amaro et al., 2021). However, researchers are emphasizing the surface properties and size of nanoparticles to overcome the resistance developed by microbes. Zheng et al. (2021) developed 4,6-diamino-2-pyrimidine thiol (DAPT)-capped gold nanoparticles (AuDAPTs), against which *E. coli* gradually developed resistance after long-term use. Though it was observed that the resistance developed was size specific, and it can be reverted by simply modifying the size of AuDAPTs. Similarly, silver covalently bound to cyanographene (GCN/Ag) effectively killed the silver nanoparticles-resistant bacteria by inducing strong interaction of GCN/Ag with the bacterial membrane (Panáček et al., 2021).

### Concentration, aggregation, and stabilization of nanoparticles

The most important parameter responsible for resistance development is the concentration of nanoparticles used and the aggregation and stabilization pattern of nanoparticles in the microbes. Recent studies found that bacteria can produce flagellin upon exposure to silver nanoparticles which can aggregate and thus deactivate the antimicrobial potential of silver nanoparticles (Panáček et al., 2018, 2021). Sub-lethal concentrations of metal nanoparticles are known to induce mutagenic effects and the emergence of selection pressure in bacterial genome (Amaro et al., 2021; McNeilly et al., 2021), though contradictory results have been reported. McNeilly et al. (2021) reported that the continuous use of AgNO_3_ and silver nanoparticles against *E. coli* resulted in Ag+ resistance development by the induction of endogenous mutation. In another study, Wu et al. (2022) reported a negligible role of sub-lethal doses of silver nanoparticles on the mutation rate and resistance development in *E. coli*.

### Presence of resistance plasmids and the conjugation rate in microbes

Many bacteria have R plasmids and metal exclusion capacity, which provides them an additional edge over metallic nanoparticles such as silver, copper, and alumina. It has been observed that the sub-lethal concentration of metallic nanoparticles can promote the conjugational transfer of multi-resistance plasmid RP-4 (Qiu et al., 2012, 2015) in certain bacterial strains and thus increases the chances of acquiring resistance against metal nanoparticles. The metal efflux complexes located in R plasmids or bacterial genome can encode proteins that are responsible for metal ion exclusion outside the bacterial cells (Salas Orozco et al., 2019).

### Biofilm-forming microbial strains

The ability to form biofilms by microbial strains is another crucial factor that decides the susceptibility or resistance to nanoparticles. Though antimicrobial nanoparticles can effectively eradicate biofilms, bacterial biofilms can also cause aggregation of nanoparticles making them ineffective as antimicrobials (Joshi et al., 2020). The components of biofilm, such as protein, lipids, nucleic acid, and polysaccharides can interact and modify the nanoparticles corona, making them lesser effective as antimicrobials (Joo and Aggarwal, 2018). Furthermore, the role of the size and surface corona of nanoparticles cannot be neglected while battling biofilms (Nallathamby et al., 2010).

### Regulation of molecular machinery by microbes

Both exogenous and endogenous molecular determinants are responsible for developing nanoparticles specific resistance in microbes. As discussed in the earlier section, mechanisms of nanoparticle resistance include multiple genes involved in oxidative stress, membrane disruption, adaptive resistance, cell division, motility, and biofilm formation. However, Panáček et al. (2021) reported that no genetic change in silver nanoparticles aggregation was involved by flagellin secretion in *E. coli*. The consecutive use of sub-lethal concentrations of metal nanoparticles in different environments and lab settings are continuously spreading the horizontal gene transfer and increases the co-selection of metallic and antibiotic resistance (Amaro et al., 2021). The studies have elucidated that DNA uptake by damaged membranes, increased mutation frequencies, and SOS DNA repair are some of the mechanisms employed by microbes after exposure to metallic nanoparticles which promote nanoparticle resistance (Crane et al., 2018; Amaro et al., 2021). It was observed that membrane alteration can act as double-edged sword for killing or developing nanoparticle resistance in bacteria. The disrupted membrane can increase the frequency of foreign DNA uptake inside the bacterial cells, which can promote the increase in conjugation frequencies and SOS DNA repair responses. The increased SOS DNA repair responses can further lead to horizontal gene transfer by increasing recombination between the foreign DNA and the recipient's DNA (Amaro et al., 2021).

It is quite evident that one single factor is not responsible for microbe resistance to nanoparticles; rather, the mechanism of microbes–nanoparticle interactions, properties of nanoparticles and microbes, and their surrounding environment decides the winner of this important battle.

## Future prospects and conclusion

Antimicrobial nanoparticles are the latest effective substitute for antibiotics for tackling multidrug resistance; however, emerging nanoparticles resistant to microbes are a cause of concern.

The use of nanoparticles as antimicrobials is relatively new, and the risk needs to be assessed carefully. For checking the unwarranted use of nanoparticles, uniform and strict regulatory authorities need to be formed and followed around the globe. Multiple drug authorities including Food and Drug Administration (FDA, USA), European Medicines Agency (Europe), and the Pharmaceuticals and Medical Devices Agency (Japan) have strict guidelines for manufacturing, uses, and nano-waste disposal which need to be followed in other countries as well.

Similar to the case of antibiotics, long-term exposure to sub-lethal doses of nanoparticles to microbes may add to the development of resistant bacteria (McNeilly et al., 2021). Several reports in laboratory settings have revealed genetic changes, mutations, and overproduction of flagellin induced by sub-lethal dosages of silver nanoparticles (Graves et al., 2015; Panáček et al., 2021). The guidelines regarding the concentration of nanoparticles used in multiple settings should be strictly followed across the countries.

Nanoparticles have now been used in every aspect, whether it is agriculture, medicine, or environment. Soil is the sink of nanoparticles used in different fields, where they further interact with different microbial communities (Yonathan et al., 2022). As previously discussed, sub-lethal dosage of nanoparticles can add to the nanoparticle resistance development in microbes; where leaching of nanoparticles to soil and water can aggravate this concern. The nanosilver present in the environmental pool can impact the antibiotic resistance determinants by co-selection (Pal et al., 2017). Multiple genes, including *silE-P* (silver resistance genes), antibiotic resistance genes, beta-lactams *(blaCTX-M)*, quinolones *(oqxAB)*, and aminoglycosides (*aac-Ib-cr*) resistance genes, were found in the plasmid of *E. coli* isolated from livestock animals (Fang et al., 2016). The co-presence of nanoparticles and antibiotic-resistance genes in the environmental samples can stimulate co-selection and nanoparticle resistance development in sensitive microbes. These situations clearly demand regulation on nanoparticle waste disposal in soil and water.

To deal with the existing problem of nanoparticle resistance developed by microbes, it is essential to understand the mechanism of acquiring nanoparticle resistance. An in-depth understanding of this recently developed phenomenon will help to reverse the resistance or modify the nanoparticles and their surface corona in a better way. For example, bacteria continuously exposed to sub-lethal concentrations of silver nanoparticles can acquire resistance by producing flagellin. By inhibiting the production of flagellin by pomegranate rind extract (PGRE), or coating the nanoparticles with PGRE, the resistance gain can be stopped (Panáček et al., 2018). Similarly, the appropriate use of nanoparticles in hospital and agricultural settings, as well as monitoring nano-waste disposal and surface manipulation of designed nanoparticles, will further help to control the nanoparticle resistance acquisition by microbes.

In conclusion, it can be confirmed that microbes, particularly, bacteria are acquiring resistance against metal nanoparticles rapidly by employing multiple mechanisms. Several factors such as concentration, aggregation, and type of nanoparticles as well as flagellin production, biofilm-forming, and presence of R plasmid in microbes can affect the resistance development in microbes. Though it is a recent phenomenon, strict regulation, adherence to the rules regarding the use and disposal of nanoparticles, and a vigilant check on nanoparticles–environment interactions are required to prevent this problem from becoming as widespread as multidrug resistance.

## Author contributions

SK and MK: investigation, formal analysis, writing—original draft, editing, and visualization. MK: conceptualization. All authors contributed to the article and approved the submitted version.

## Footnotes

^1^*Sustaining Action Against Antimicrobial Resistance: A Case Series of Country Experiences*. Available online at: https://www.who.int/news/item/17-10-2022-sustaining-action-against-antimicrobial-resistance-a-case-series-of-country-experiences (accessed November 6, 2022).
